# Electroacupuncture suppresses glucose metabolism and GLUT-3 expression in medial prefrontal cortical in rats with neuropathic pain

**DOI:** 10.1186/s40659-021-00348-0

**Published:** 2021-08-06

**Authors:** Menghong Jiang, Xiaomei Chen, Liangping Zhang, Weiting Liu, Xiangmei Yu, Zhifu Wang, Meifeng Zheng

**Affiliations:** 1 Fujian University of Traditional Chinese Medicine, Fuzhou, 350122 Fujian China; 2 Key Laboratory of Orthopedics & Traumatology of Traditional Chinese Medicine and Rehabilitation, Fujian University of Chinese Medicine Affiliated Rehabilitation Hospital, Fuzhou, 350122 Fujian China

**Keywords:** Electroacupuncture, Neuropathic pain, Medial prefrontal cortex, Glucose metabolism, Glucose transporter-3

## Abstract

**Background:**

Accumulating evidence has demonstrated that the electroacupuncture (EA) stimulation could effectively alleviate neuropathic pain. The medial prefrontal cortex (mPFC) is a vital part of the cortical representation of pain in the brain, and its glucose metabolism is mostly affected in the progression of pain. However, the central mechanism of EA analgesia remains unclear.

**Methods:**

Fifty-four male SD rats were equally randomized into sham surgery (Sham) group, chronic constriction injury (CCI) group and EA stimulation (EA) group. The CCI model, involving ligature of the right sciatic nerve, was established in all animals except the Sham group. EA stimulation was applied on the right side acupoints of Huantiao (GB30) and Yanglingquan (GB34) in the EA group. Paw withdrawal threshold (PWT) and paw thermal withdrawal latency (PWL) were measured. The ^18^ F-fluorodeoxyglucose positron emission tomography (FDG-PET) was used to evaluate glucose metabolism changes in the mPFC. The expression of glucose transporter 3 (GLUT-3) in the mPFC was determined by immune histochemistry and ELISA.

**Results:**

Comparing with CCI groups, EA treatment was obviously reversed CCI-induced mechanical allodynia (P < 0.01), thermal hyperalgesia (P < 0.01) and the increase of glucose metabolism in the left mPFC (P < 0.05). Furthermore, EA treatment significantly decreased the protein expression of GLUT-3 in the left mPFC (P < 0.01).

**Conclusions:**

Our results indicate that EA analgesia effect may be related to suppressing the glucose metabolism and GLUT-3 expression in the mPFC. This study could provide a potential insight into the central mechanisms involved in the analgesic effect of EA.

## Introduction

Neuropathic pain (NP), an extremely severe chronic condition, is stimulated or caused by the primary lesion or dysfunction of the central or peripheral nervous system with very complex pathological changes [1]. Considered as a public health problem, NP affects as high as 6.9–10% of the general population [2]. The worldwide clinical practice and experimental studies have shown that acupuncture can effectively relieve neuropathic pain symptoms without producing significant side effects [3–6]. However, the central mechanism of electroacupuncture in the treatment of NP still needs to be further explored.

It is well known that glucose metabolism is the dominant energy provider for the brain; that is, the cerebral glucose metabolism reflects the brain state to some extent [7]. Functional neuroimaging studies have shown that glucose metabolism changes occur in the critical brain regions such as thalamus [8], cerebellum [9], primary somatosensory cortex [10], insular cortex [11], anterior cingulate cortex, and prefrontal cortex (PFC) during the neuropathic pain [12, 13]. More researches have shown that altered neuronal morphology, altered neuronal excitability and glucose metabolism in the medial prefrontal cortex (mPFC) play vital roles in the development of neuropathic pain. Increased spine number and NMDA currents in the mPFC may lead to increased glutamatergic input-mediated calcium influx, glutamate excitotoxicity and neuronal loss [14–18]. Injected a partial agonist of NMDA receptor into mPFC could induce antinociception in the spared nerve injury (SNI) rats, while antinociception by motor cortex stimulation would suppress the BOLD signals in the mPFC [19, 20]. Besides, classical anti-neuralgia drug gabapentin could relieve pain response by decreasing the glucose metabolism of mPFC [12]. Transcranial direct current stimulation (tDCS) to the neuropathic pain patients could induce decreased glucose metabolism in the prefrontal cortex [13].

Glucose transporter-3 (GLUT-3), the major neuronal glucose transporter, is closely associated with local cerebral glucose utilization in different brain regions [21]. The up-regulation of GLUT-3, both in mRNA and protein expression of the dorsal root ganglia (DRG), was observed in the neuropathic pain rats. VitC can enhance the analgesic effect of gabapentin in CCI rats via reducing the GLUT-3 expressing [22]. In the clinical treatment and rodent study of neuropathic pain, electroacupuncture with Huantiao (GB30) and Yanglingquan (GB34) acupoints played significant analgesic effect [23–25]. Some researches had shown that electroacupuncture could induce increased or decreased glucose metabolism of some brain regions in chronic pain [26, 27]. Interestingly, our previous studies found that electroacupuncture can elevate cerebral glucose metabolism and GLUT-3 expression in cognitive impairment mice [28]. However, in the state of neuropathic pain, whether electroacupuncture can play an analgesic role by regulating the changes of cerebral glucose metabolism and GLUT3 expression remains to be further studied.

According to all above literature, we hypothesized that EA treatment alleviated neuropathic pain by decreasing the glucose metabolism and the expression of GLUT-3 in the mPFC. In the present study, we assessed the analgesic effect of EA in the chronic constriction injury (CCI) rats and explored the underlying central mechanisms.

## Material and methods

### Animals and group of experiments

Adult male Sprague–Dawley rats (200 ± 20 g) from The Shanghai SLAC Laboratory Animal Co., Ltd (Shanghai, China) were used. The animals were housed in groups of 5 per cage with free access to water and food, on a 12-h light/dark cycle (lights on/off 06:00/18:00 h) in a temperature-controlled environment (20 ± 2 °C). After adaptive feeding 7 days, rats were randomized into three groups, including sham surgery group (Sham, n = 18), CCI model group (CCI, n = 18) and EA stimulation group (EA, n = 18). Eight rats were used for PET/CT scans, six for immunohistochemistry and four for ELISA from each group. All experimental procedures were approved by the Ethics Committee of Fujian University of Traditional Chinese Medicine (Fuzhou, China) and all efforts were made to minimize the number of animals used and their suffering.

### CCI model

All the rats underwent surgery for chronic constriction injury according to Bennett and Xie [29], except for the sham group. Briefly, 2% inhaled isoflurane via a precision vaporizer was used for animal anaesthesia maintenance. The right sciatic nerve was exposed at the midthigh level, then four 4–0 chromic gut ligatures were loosely tied around with about 1 mm apart. Sham group animals underwent the same procedure without the ligation.

### Electroacupuncture

**Fig. 1:**
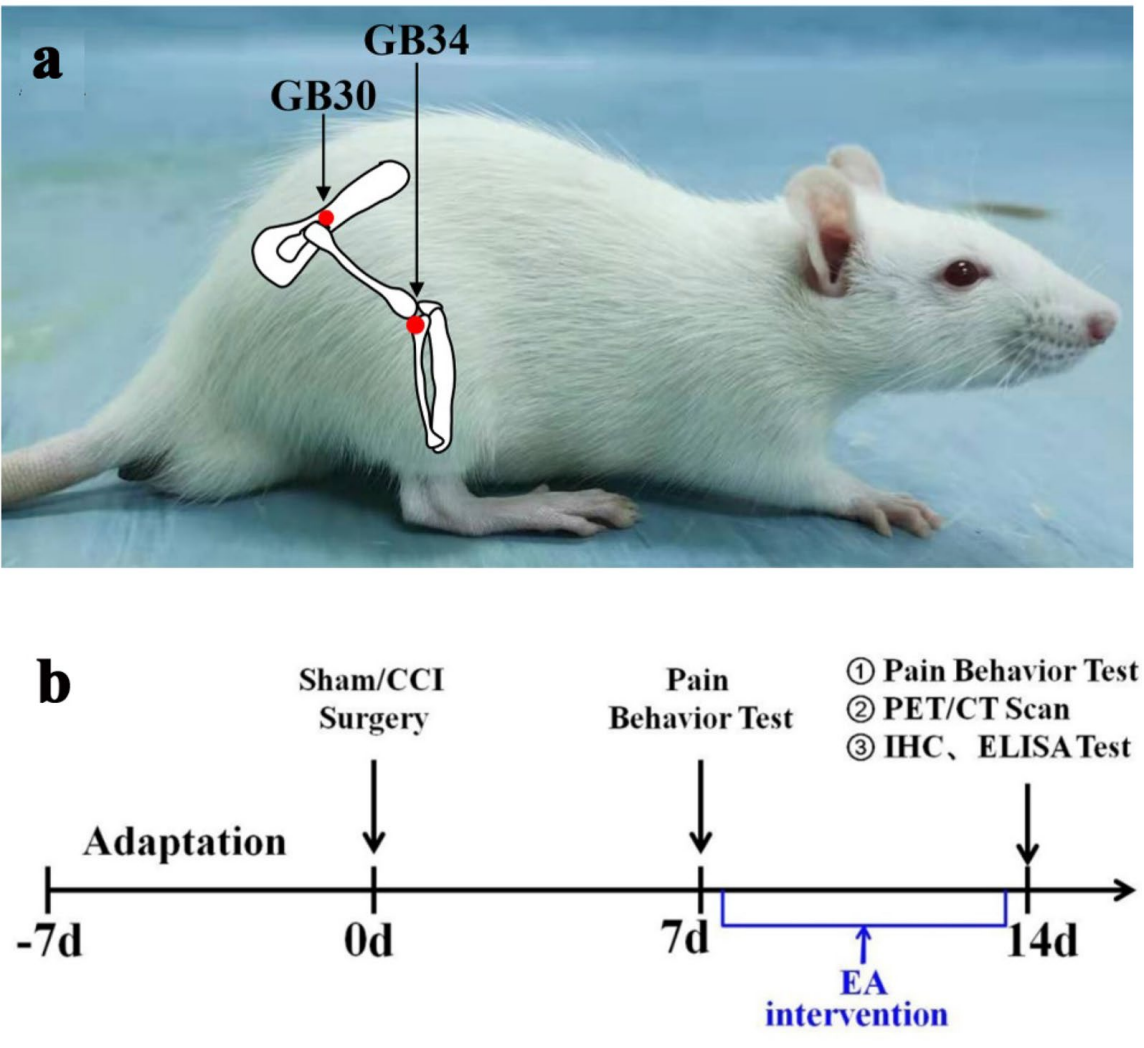
**a** Localization of acupoints used by electroacupuncture. **b** Flow chart of the experiment

The Huantiao (GB30) acupoint was located at the posterior upper border of the hip joint of the hindlimbs. Yanglinquan (GB34) was located near the knee joint, anterior and inferior to the capitula fibula, in the peroneus longus and brevis muscle (Fig. 1A). The right side of points in EA group rats were inserted into with Huatuo needles (Mode: 0.25 × 13 mm, Suzhou Medical Appliance Factory, Suzhou, China) at a depth of approximately 5 mm. From the 7th postoperative day, the animals in EA group underwent EA stimulation with 2 Hz frequency at 1 mA for 30 min (SDZ-II Huatuo Electroacupuncture Instrument, Suzhou Medical Appliances Co., Ltd., Suzhou, China), once daily for seven consecutive days (Fig. 1B). During EA stimulation, the animals were constrained with a special cloth bag, kept calm and awake, without obvious stress. The other two groups underwent the same procedure except for acupuncture and EA stimulation.

### Assessment of mechanical and thermal hyperalgesia

On the 0, 7th and 14th day after surgery, the mechanical and thermal allodynia of right hind paw in all rats were measured (Fig. 1B). Before the test started, rats were habituated in separate transparent Plexiglas cages (size:20 cm × 20 cm × 20 cm) with wire mesh floor (grid size:0.5 cm × 0.5 cm) for 15 min. Fifty percent paw withdrawal thresholds (50% PWTs) were assessed with the up-down method according to previous research [30]. Each von Frey hair was held to the plantar surface of the right hind paw for 2–3 s, with a 30 s interval between stimuli. Positive responses to the von Frey hair stimulation include quick withdrawal, licking and/or shaking of the paw.

Paw withdrawal latencies (PWLs) were obtained by using a focused radiant heat to stimulate the sole of right hind paw with a thermal measurement instrument (Type PL-200, Chengdu Tai League Software Co., Ltd., Chengdu, China). A 20 s cut-off was set up for avoiding tissue damage. Only quick withdrawal of the right hind paw was defined as a positive response. After collected five times with an interval of at least 5 min, PWLs were averaged as the thermal threshold for pain.

### PET/CT scans

On the 7th and 14th day after surgery, eight rats were randomized from each group for PET/CT scans, with Micro-PET/SPECT/CT imaging system (Milabs, Netherlands). In order to avoid the affection of the 18F-FDG biodistribution, rats fasted overnight before scanning. Animals were intraperitoneally injected with 18F-FDG as a radioactive tracer with dose of 40 MBq. After immediate pain behavior detection, rats were put into an anaesthesia induction box (5% isoflurane, oxygen flow rate 400 ml/min) for 5 min. During scanning, the rats were stabilized with cellophane tape in the prone position, maintained low concentration anaesthesia (1% isoflurane, oxygen flow rate 250 ml/min). After acquired the images in 3D mode, we reconstructed CT images with filter-back projection algorithm and PET images with pixel-based ordered subsets expectation maximization algorithm. All the PET/CT images were analyzed and fused with the Rat W.Schiffer Brain Atlas in the coronal plane, sagittal plane and horizontal plane, using PMOD software (PMOD Technologies, Zurich, Switzerland), and three-dimensional regions of interest were selected in the mPFC. Finally, the percentage injected dose per gram (% ID/g) of the mPFC was obtained with the PMOD software and for subsequent quantitative analysis.

### Immunohistochemistry

Two weeks after surgery, six rats were randomized from each group for immunohistochemistry. The anesthetized rats were perfused intracardially with 0.9% saline solution followed by a 4% paraformaldehyde (PFA) solution. The rat's brain were soaked with 4% PFA and the paraffin embedded. After cut into 5 μm thick, the tissue sections were deparaffinized in xylene and rehydrated in graded ethanol and water. Antigen retrieval was performed at a antigen repair solution (pH 9.0) using a pressure cooker for 2 min. To block endogenous peroxidase activity, the slides were immersed in 3% hydrogen peroxide away from light at room temperature (RT) for 25 min, followed by three washes, then blocked with 5% bovine serum albumin (BSA) at RT for 30 min. Anti-GLUT-3 antibody (1:50, Abcam) was added and incubated with sections overnight at 4 ºC, then secondary antibodies were used HRP-labeled rabbit anti-goat (Abcam) at RT for 50 min. The color was developed with freshly prepared diaminobenzidine chromogenic solution, counterstained with hematoxylin. Finally, rinse slides, dehydrate through ascending concentrations of alcohol, clear in xylene and mount. The sections were observed under a DM4000B microscope (Leica, Wetzlar, Germany) at 400 × magnification, and images were obtained in the PrL region in the mPFC according to the stereotaxic atlas of the rat brain. The positive staining of GLUT-3 was brownish yellow. The positive cells in five randomly selected fields were evaluated. The average optical density (AOD) of GLUT-3 were detected by using Image-Pro plus 6.0 software for quantitative analysis.

### ELISA

Two weeks after surgery, four rats from each group were used for ELISA. Rats were euthanized and the mPFC in brain was rapidly removed and isolated on ice, kept frozen at − 80 °C. The expression of GLUT-3 in the left mPFC was measured using ELISA kits (Jiangsu Meimian industrial Co., Ltd, Jiangsu, China), following the manufacturer protocol. The absorbance peak (OD value) was measured using a microplate reader at the wave length of 450 nm. The results were shown as micrograms per milliliter (µg/ml).

### Statistics

Analyzed by SPSS 21.0 software (IBM, Chicago, IL, USA), data are expressed as mean ± standard deviation. Analysis of variance (ANOVA) was carried out in case of normal distribution. P < 0.05 was regarded as a significant difference. Results were graphically presented using Graph Pad Prism 8 software.

## Result

### EA treatment alleviated the CCI-induced mechanical allodynia and thermal hyperalgesia

**Fig. 2:**
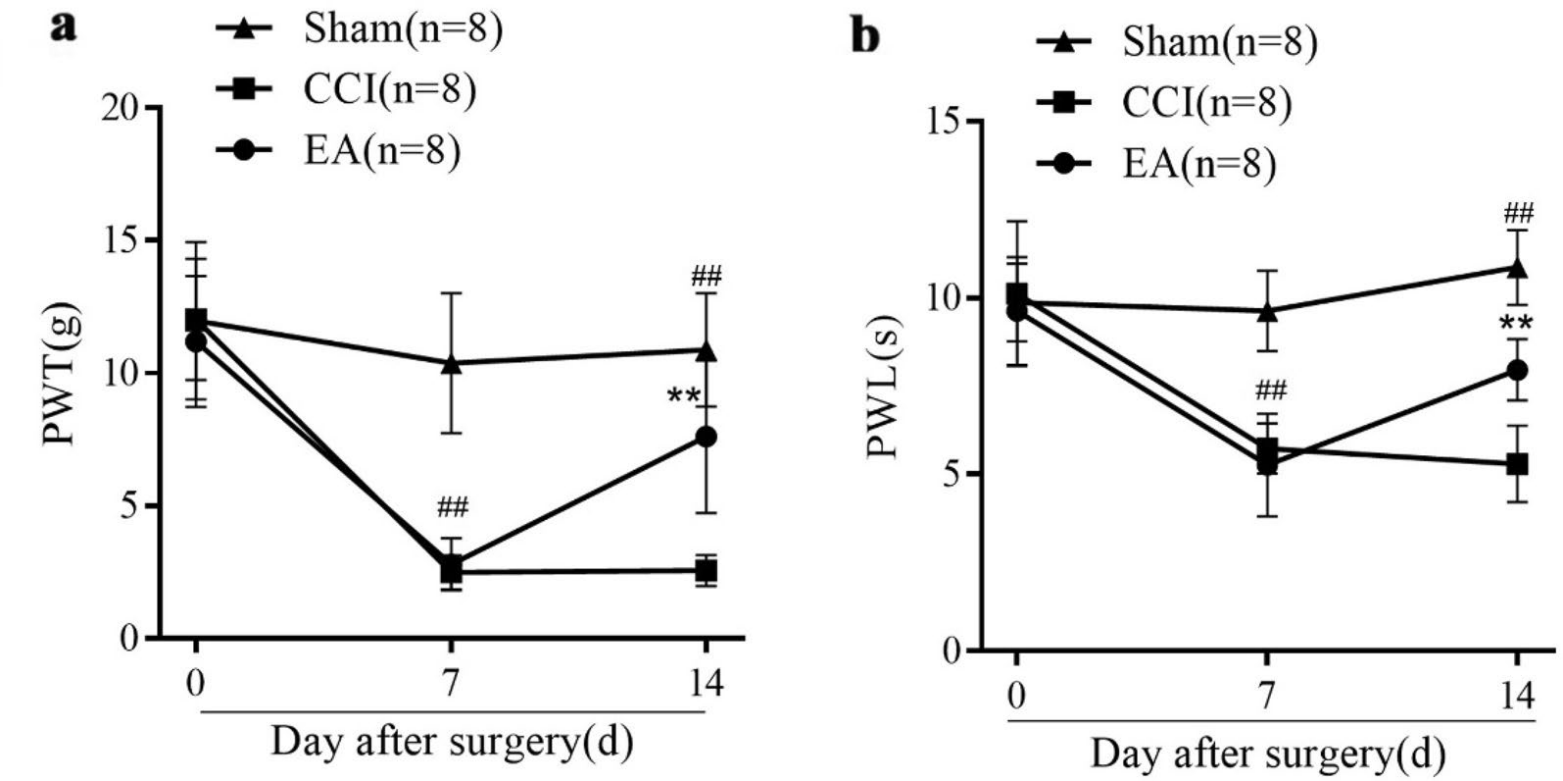
Effects of EA treatment in PWT and PWL of rats from the Sham, CCI and EA groups (n = 8 per group). PWT (**a**) and PWL (**b**) were recorded on the 0, 7th and 14th day after surgery. Data are presented as mean ± SD. Post hoc Tukey test ^*##*^* P* < 0.01, compared with the Sham group; **P < 0.01, compared with the CCI group

There was no difference in the baseline PWT and PWL between three groups before CCI surgery (*P* > 0.05, Fig. 2a, b). Compared with the Sham group, CCI surgery induced markedly PWT and PWL decrease in rats from CCI and EA groups on the 7th day (^*##*^* P* < 0.01). After one week EA stimulation, a significant PWT and PWL increase were observed in the EA group (**P < 0.01, Fig. 2a, b), suggesting the obvious EA-induced analgesic effect.

### EA treatment reversed CCI-induced increase of glucose metabolism in the left mPFC

**Fig. 3:**
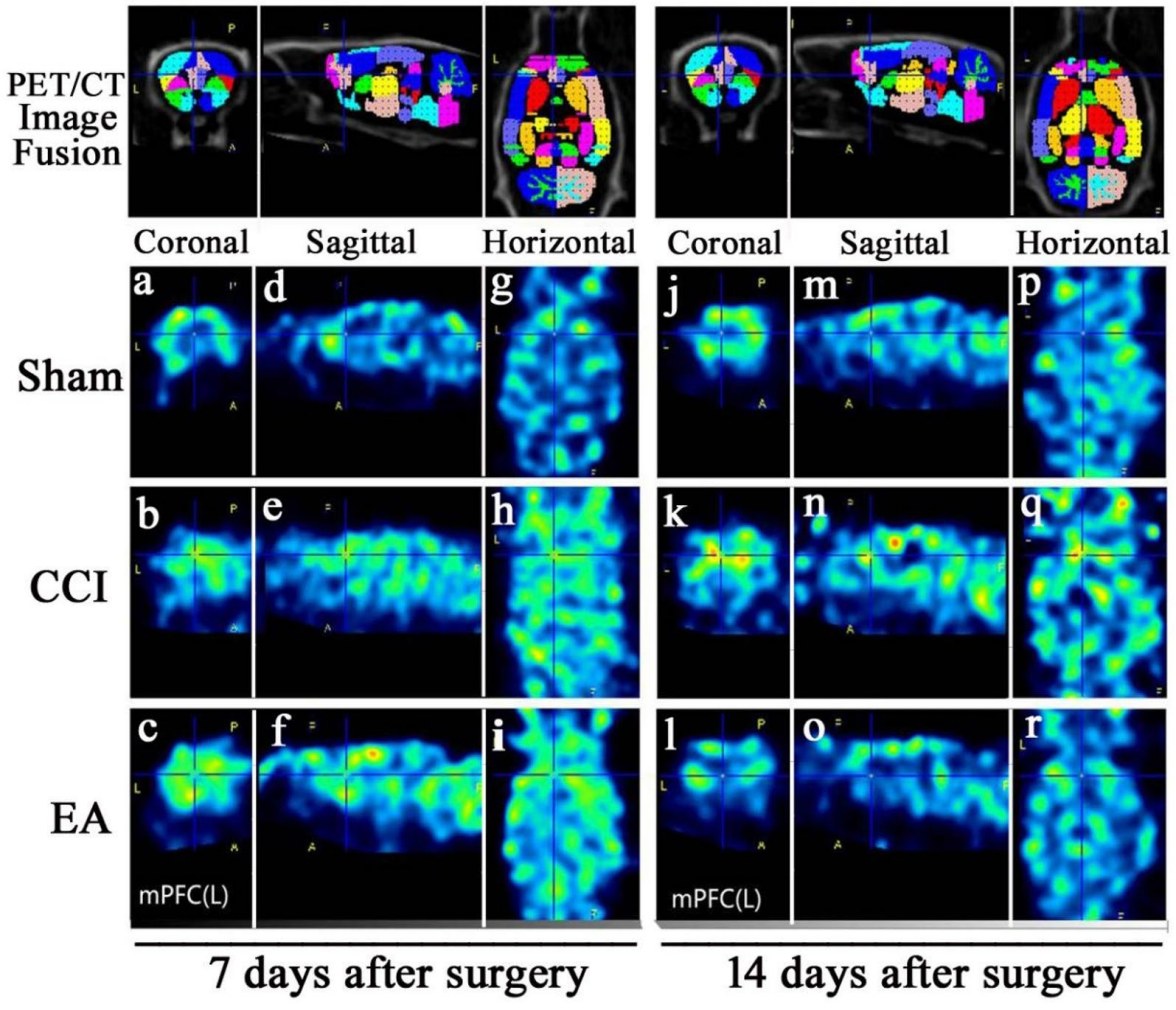
Before and after EA stimulation, 18F-FDG/PET/CT images of the left mPFC in rats from three groups. PET/CT image fusion: Fig **a**, **b**, **c**, **j**, **k**, **l** (coronal plane); Fig **d**, **e**, **f**, **m**, **n**, **o** (sagittal plane); Fig **g**, **h**, **i**, **p**, **q**, **r** (horizontal plane). Colour codes: Red = high, Blue = low

**Fig. 4:**
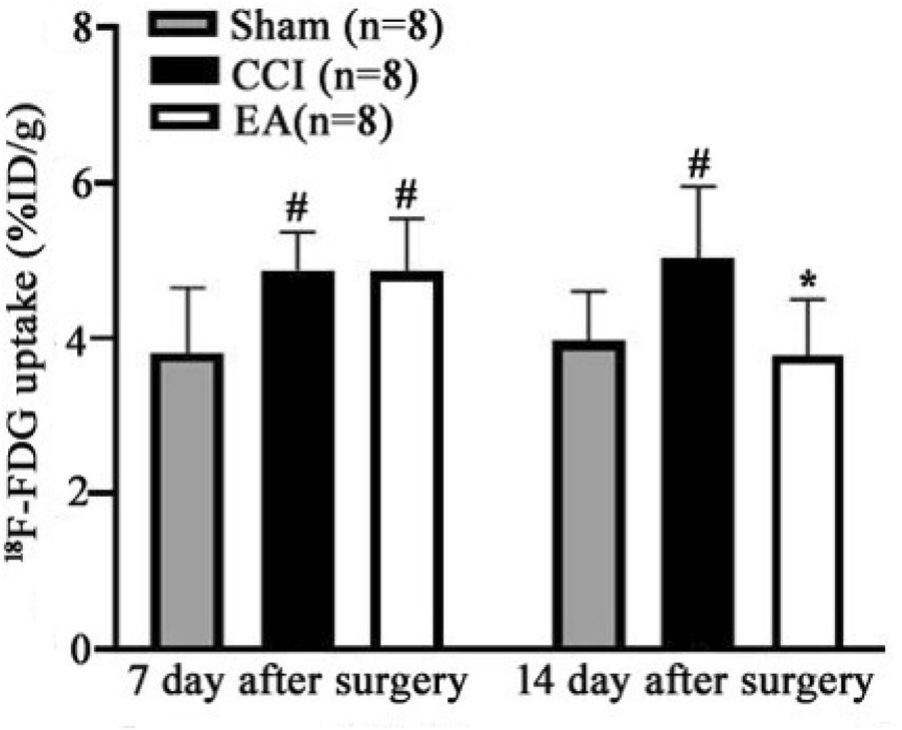
Before and after EA stimulation, the uptake rates in the left mPFC of rats from three groups (n = 8 per group). Post hoc Tukey test, ^#^*P* < 0.05, compared with the Sham group; ^*^P < 0.05, compared with the CCI group

The present study used ^18^F-FDG images to show the changes of glucose metabolism in the left mPFC from all rats (Fig. 3). With the same color standard, the color code indicates glucose metabolism ranging from blue (low expression) to red (high expression). Compared CCI and EA groups with Sham group, the uptake rates of the left mPFC were increased on the 7th day after surgery without EA stimulation (^*#*^*P* < 0.05, Fig. 4). However, compared with the CCI group, the uptake rates in EA group rats were decreased on the 14th day after surgery (*P < 0.05, Fig. 4).

### EA treatment decreased the GLUT-3 expression in the left mPFC of CCI rats

**Fig. 5:**
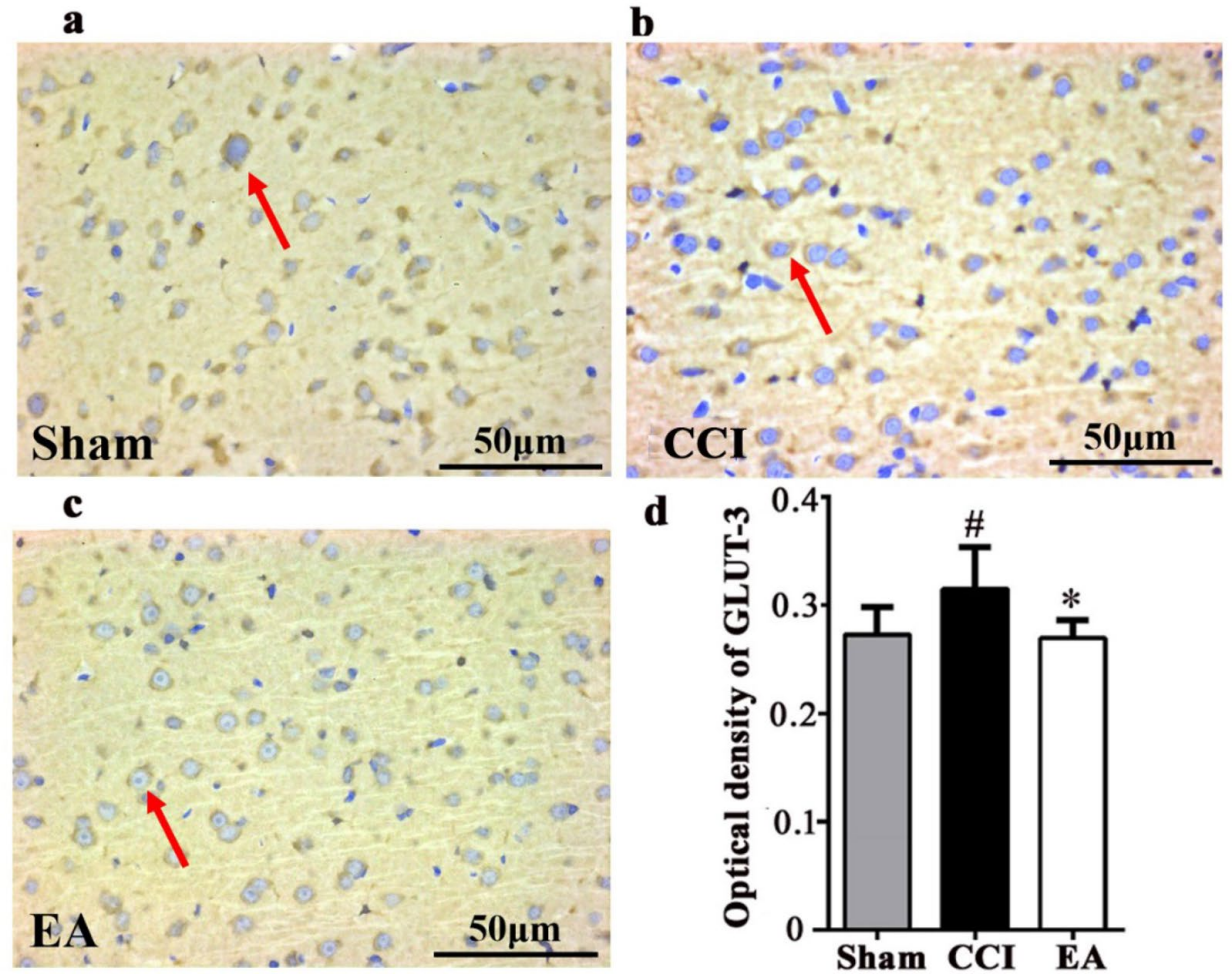
The representative image and average optical density of GLUT-3 by the immunohistochemical detection in the left mPFC in rats from three groups. Representative image (Scale bar: 50 μm) of IHC staining of GLUT-3 in the left mPFC of rats from **a** Sham group (n = 6), **b** CCI group (n = 6), **c** EA group (n = 6). Positive immunoreactivity appears as brown color, and the GLUT-3 immunoreaction product was mainly expressed in the cell membrane (red arrow). **d** Post hoc Tukey test^*#*^* P* < 0.05, compared with the Sham group. ^*^P < 0.05, compared with the CCI group

The GLUT-3 immunoreaction product was visible under the light microscope, represented as brown color in the Fig. 5a–c. Immunohistochemistry results demonstrated that, compared with the Sham group, the GLUT-3 expression levels in the left mPFC of rats from CCI group were significantly increased (^*^P < 0.05, Fig. 5d). However, after EA intervention, the GLUT-3 expression levels in the left mPFC of rats from EA group were decreased compared with the CCI group (^#^*P* < 0.05, Fig. 5d).

**Fig. 6:**
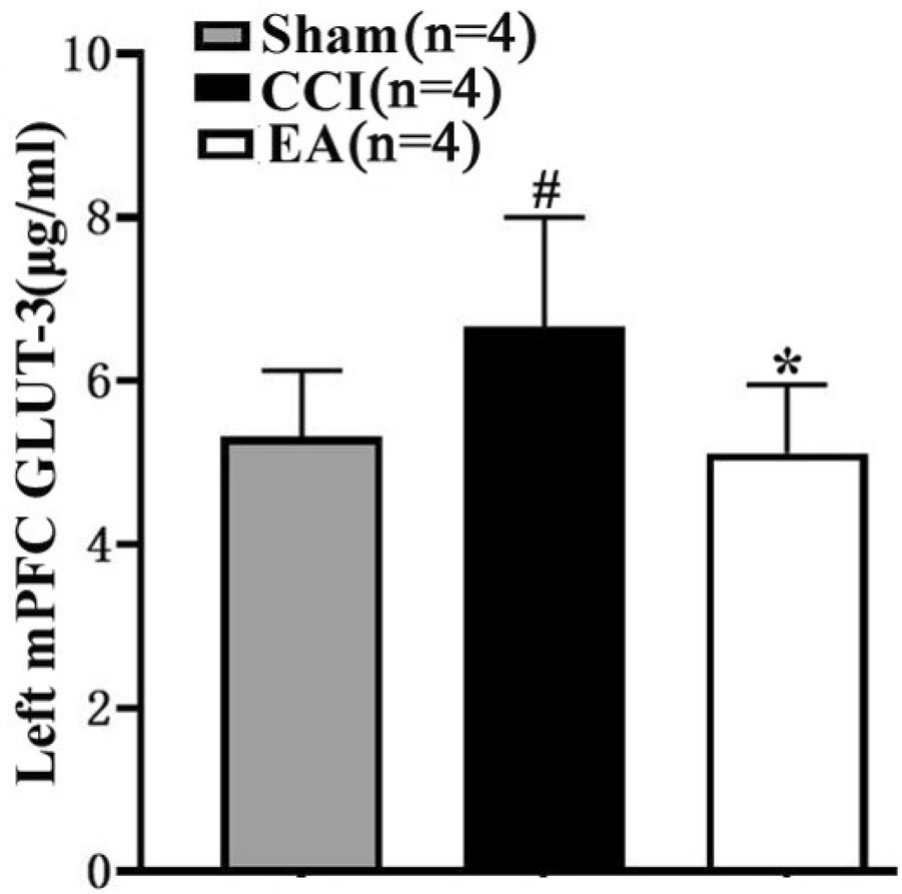
GLUT-3 concentrations on the left mPFC detected by ELISA in three groups (Sham, n = 4; CCI, n = 4; EA, n = 4). Post hoc Tukey test, ^#^*P* < 0.01, compared with the Sham group, *P < 0.01, compared with the CCI group

To further investigate the influence of EA on GLUT-3 changes in the left mPFC, ELISA detection was used to test the concentrations of GLUT-3 in the left mPFC. Compared with the Sham group, the concentrations of GLUT-3 in the left mPFC of rats from CCI group were markedly increased (^*^P < 0.01, Fig. 6). On the other hand, the concentrations of GLUT-3 in the mPFC of rats from EA group were decreased compared with the CCI group (^#^*P* < 0.01, Fig. 6).

## Discussion

EA is combined traditional acupuncture with electrical stimulation and widely used for analgesia of various neuropathic pain conditions. Recent clinical researches have shown that EA treatment with GB30 and GB34 presents an obvious analgesic effect towards sciatic pain patients [23]. In animal experiments, EA stimulation at acupoints GB30 or GB34 can alleviate both allodynia and hyperalgesia by regulating neurotransmitters and receptors, cell factors or synaptic function [31–33]. In our experiment, we first used the glucose metabolism of mPFC to evaluate the analgesic effect of EA in GB30 and GB34 acupoints, also to explore the superior central central mechanism.

Chronic pain can cause the activation in pain-related brain regions such as prefrontal cortex (PFC), anterior cingulate cortex (ACC), insula, thalamus [34]. Recently more and more literature are focused on the mPFC function in the process of pain states via PET or fMRI detection. At the brain functional level, hyper- and hypoactivity in the mPFC were both observed in chronic pain patients [17, 35]. tDCS or spinal manipulation therapy (SMT) intervention could relieve patients' pain by reducing glucose metabolism in the PFC [13, 36]. Gabapentin can decrease glucose metabolism in the mPFC to reverse central hypersensitivity [12]. In addition, some research suggested that the effects of EA treatment could reduce the mechanical allodynia by inhibiting the TRPV1 receptor activation of mPFC during chronic pain. EA also could reverse the decreasing NMDA signaling pathway of PFC during pain and depression [37, 38]. Decreased activated voxel values were observed in the PFC of visceral pain patients after consecutive EA stimulation [39, 40]. Local combined distal EA produced deactivation in the medial prefrontal cortex (PFC) in Carpal Tunnel Syndrome patients [41]. In our research, decrease glucose metabolism in the mPFC were found after EA stimulation in CCI rats, which maybe the different pain regulatory pathway compared with TRPV1 receptors and NMDA signaling pathways.

In order to further explore the specific mechanism of glucose metabolism in EA treatment with neuropathic pain, we detected and analyzed the GLUT3 of mPFC. GLUT3, one of the major glucose transporters in the brain, has a high affinity for glucose and a great capacity for ensuring efficient glucose transport across cell membranes, thus its expression levels of different regions are related to local glucose utilization [21]. The GLUT-3 protein levels in the PFC was significantly decreased in cognitive impairment and schizophrenia [42, 43]. Previous studies found that the changes of GLUT-3 expression in the dorsal horn of the spinal cord seem to depend on different pain models. GLUT-3 was up-regulated in the SNI animals, and mainly expressed in astrocytes and microglia cells [22, 44]. However, the GLUT-3 expression level was unchanged in sciatic nerve transection of the frogs [45]. Our team studies have pointed out that, EA treatment increased brain regional glucose metabolism and the expressions of GLUT-3 in neurons of the hippocampus and cortex, consequently alleviating cognitive impairment [28]. In our present study, the upregulated expression of GLUT-3 in the left mPFC were markedly suppressed in CCI rats after EA treatment. We speculated that glucose metabolism and Glu3 expression would change differently in various brain regions under common disease states (such as memory impairment and chronic pain).

Some studies have confirmed that during the CCI induced neuropathic pain, the expression of spinal reactive oxygen species (ROS) and GLUT3 were increased, along with the increase in dehydroascorbate (DHA) production, which is closely related to the occurrence of central sensitization [22, 46]. In fact, a link between neuropathic pain and oxidative stress at supraspinal levels such as PFC had been demonstrated. However, in our study, there is still insufficient evidence to show that electroacupuncture inhibition of PFC glucose metabolism is related to antioxidant stress during neuropathic pain [47].

Although GLUT-3 is highly and specifically expressed by neurons, recent evidence indicates that it was expressed in neuroglia under stress conditions, such as hypoxia and ischemia [48]. Our future work will pay attention to the localization of GLUT-3 expression and injected with GLUT-3 antagonist into the mPFC, so as to further clarify the mechanism. In addition, we would need to further explore the relationship between electroacupuncture regulation of glucose metabolism and central sensitization in the mPFC.

## Conclusions

Our results indicate that EA analgesia effect may be related to suppressing the glucose metabolism and GLUT-3 expression in the mPFC. This study could provide a potential insight into the central mechanisms involved in the analgesic effect of EA. Further studies investigating the effects of EA on neuropathic pain are required to verify our conclusions.

## Data Availability

Not applicable.

## Abbreviations

ACC: Anterior cingulate cortex
ANOVA: Analysis of variance
CCI: Chronic constriction injury
CFA: Complete Freund’s adjuvant
DRG: Dorsal root ganglia
EA: Electroacupuncture
fMRI: Functional magnetic resonance imaging
GLUT-3: Glucose transporter 3
mPFC: Medial prefrontal cortex
NP: Neuropathic pain
NMDA: N-methyl-D-aspartic acid receptor
PWT: Paw withdrawal threshold
PWL: Paw thermal withdrawal latency
tDCS: Transcranial direct current stimulation
TRPV1: Transient receptor potential vanilloid-1
SMT: Spinal manipulation therapy
DHA: Dehydroascorbate
ROS: Reactive oxygen species
AA: Ascorbic acid
